# A Phage-Like Plasmid Carrying *bla*_KPC-2_ Gene in Carbapenem-Resistant *Pseudomonas aeruginosa*

**DOI:** 10.3389/fmicb.2019.00572

**Published:** 2019-03-20

**Authors:** Renata Galetti, Leonardo Neves Andrade, Alessandro M. Varani, Ana Lúcia Costa Darini

**Affiliations:** ^1^School of Pharmaceutical Sciences of Ribeirão Preto, University of São Paulo, Ribeirão Preto, Brazil; ^2^School of Agricultural and Veterinary Studies, São Paulo State University “Julio de Mesquita Filho” (UNESP), Jaboticabal, Brazil

**Keywords:** transduction, transposition, carbapenemases, plasmid, bacteriophages

## Abstract

**Background:** Lateral gene transfer plays a central role in the dissemination of carbapenem resistance in bacterial pathogens associated with nosocomial infections, mainly *Enterobacteriaceae* and *Pseudomonas aeruginosa*. Despite their clinical significance, there is little information regarding the mobile genetic elements and mechanism of acquisition and propagation of lateral genes in *P. aeruginosa*, and they remain largely unknown.

**Objectives:** The present study characterized the genetic context of *bla*_KPC-2_ in carbapenem-resistant *P. aeruginosa* strain BH9.

**Methods:** *Pseudomonas aeruginosa* BH9 sequencing was performed using the long-read PacBio SMRT platform and the Ion Proton System. *De novo* assembly was carried out using the SMRT pipeline and Canu, and gene prediction and annotation were performed using Prokka and RAST.

**Results:** *Pseudomonas aeruginosa* BH9 exhibited a 7.1 Mb circular chromosome. However, the *bla*_KPC-2_ gene is located in an additional contig composed by a small plasmid pBH6 from *P. aeruginosa* strain BH6 and several phage-related genes. Further analysis revealed that the beginning and end of the contig contain identical sequences, supporting a circular plasmid structure. This structure spans 41,087 bp, exhibiting all the Mu-like phage landmarks. In addition, 5-bp direct repeats (GGATG) flanking the pBH6 ends were found, strongly indicating integration of the Mu-like phage into the pBH6 plasmid. Mu phages are commonly found in *P. aeruginosa*. However, for the first time showing a potential impact in shaping the vehicles of the dissemination of antimicrobial (e.g., plasmid pBH6) resistance genes in the *Pseudomonas* genus.

**Conclusion:** pBH6 captured the Mu-like Phage BH9, creating a co-integrate pBH6::Phage BH9, and this phage-plasmid complex may represent novel case of a phage-like plasmid.

## Introduction

Carbapenem resistance mediated by the production of *Klebsiella pneumoniae* carbapenemase (KPC) enzymes has been reported worldwide in *Enterobacteriaceae* and *Pseudomonas aeruginosa*, and the *bla*_KPC-2_ gene has been found to be associated with different transposon families and plasmids (Galetti et al., 2016; Naas et al., 2016). Chromosomal mutations and horizontal gene transfer (conjugation, transformation, and transduction) have been found to be the genetic events responsible for the selection and dissemination of antimicrobial resistance (Naas et al., 2016). Transduction is genetic exchange mediated by independently replicating bacterial viruses called bacteriophages, or phages. These genetic elements are frequently associated with the dissemination and persistence of virulence genes and pathogenicity islands in primary human pathogens and foodborne pathogens (Marti et al., 2014; Colavecchio et al., 2017a; Pallen and Wren, 2007). Although phages have been found to be related to the spread of antimicrobial resistance genes (Muniesa et al., 2004; Rolain et al., 2011; Balcazar, 2014; Jaque et al., 2018), there are no reports of them as surrounding or related to carbapenemase-encoding-genes in the *Pseudomonas* genus. The aim of the present study was to analyze and characterize the genetic context of *bla*_KPC-2_ in a *P. aeruginosa* isolate from a Brazilian hospital outbreak.

## Materials and Methods

### Bacterial Isolate

Carbapenem-resistant *P. aeruginosa* BH9 (hereafter referred as BH9) was isolated in Belo Horizonte city, southeast of Brazil, from a nasal swab of an inpatient in the ward of a tertiary medical care center in 2011. Minimum inhibitory concentration (MIC) values for ceftazidime, cefepime, aztreonam, imipenem, and meropenem were determined by Etest^®^ (bioMérieux, France). The MIC of polymyxin B was determined using the microdilution broth method, and interpreted according to the Clinical and Laboratory Standards Institute (CLSI) (Clinical and Laboratory Standards Institute [CLSI], 2013). The BH9 sequence type was determined using the multilocus sequence typing (MLST) protocol^1^ and the MLST 1.8 web tool from the Center for Genomic Epidemiology^2^.

### Whole Genome Sequencing and Data Analyses

Whole genome sequencing of *P. aeruginosa* BH9 was performed using the long-read PacBio SMRT sequencing (Pacific Bioscience, CA, United States) platform and the Ion Proton System (Thermo Fisher Scientific, MA, United States). *De novo* assembly was carried out using a combination of SMRT Analysis Software v2.3.0 and Canu (Koren et al., 2017). In addition, two iterations of the quiver step from the SMRT pipeline were carried out. Due to the higher error rates commonly observed in the PacBio sequencing, the CLC Genomics Workbench v9 was used to map the Ion Proton reads to the PacBio assembly, correcting indels and low-quality regions and generating the final polished assembly. Gene prediction was performed with the Prokka pipeline (Seemann, 2014) and RAST Server (Aziz et al., 2008). The annotation of the *P. aeruginosa* BH9 Mu-like phage and *bla*_KPC-2_ was manually curated using BLASTN and BLASTP searches. Web tools provided by the Center for Genomic Epidemiology^3^ were used to predict antimicrobial resistance genes (ResFinder) (Zankari et al., 2012) and plasmid incompatibility groups (PlasmidFinder) (Carattoli et al., 2014), and the PHASTER web server was used to predict prophage regions in the genome of BH9 (Arndt et al., 2016). The antimicrobial resistance genes were predicted using ResFinder 3.1^4^.

## Results and Discussion

*Pseudomonas aeruginosa* BH9 isolate was characterized as a multidrug-resistant (MDR) bacteria. The MIC values were ≥256 μg/mL for imipenem, meropenem, ceftazidime, aztreonam, and cefepime, and 1.5 μg/mL for polymyxin B. In addition, BH9 belongs to ST381, and this ST was related to *P. aeruginosa* isolated from hospital effluent in 2010 in Brazil and from other sources in different countries, including Spain, France, Australia, and Ivory Coast (ST-381 entry from PubMLST database^1^).

*Pseudomonas aeruginosa* BH9 PacBio sequencing yielded 54,168 reads, with 26 kb of N50 (mean 15 kb) and a mean read score of 0.84, generating a total of 821,031,951 bp. In addition, 33 M of Ion Proton single-end reads with an average length of 140 bp were generated and used to polish the final assembly. The *P. aeruginosa* BH9 assembly was contained in a 7.1 Mb circular genome, with GC of 65.68%, encoding to 6,733 CDSs (139 pseudogenes), 75 tRNAs, 1 tmRNA, and four copies of the rRNA operon, and one additional contig containing the *bla*_KPC-2_ gene (see below). The final polished assembly exhibited 90× and 650× of PacBio and Ion Torrent coverage, respectively.

The *P. aeruginosa* BH9 resistome consists in seven different antimicrobial resistance genes: *bla*_KPC-2_, *bla*_OXA-50_, *bla*_PAO_ (beta-lactam resistance), *aph(3′)-IIb* (aminoglycosides resistance), 2 copies of *crpP* (fluoroquinolone resistance), *catB7* (phenicol resistance), and *fosA* (fosfomycin resistance). All the antimicrobial resistance genes were located in the bacterial main chromosome, except *bla*_KPC-2_.

**FIGURE 1:**
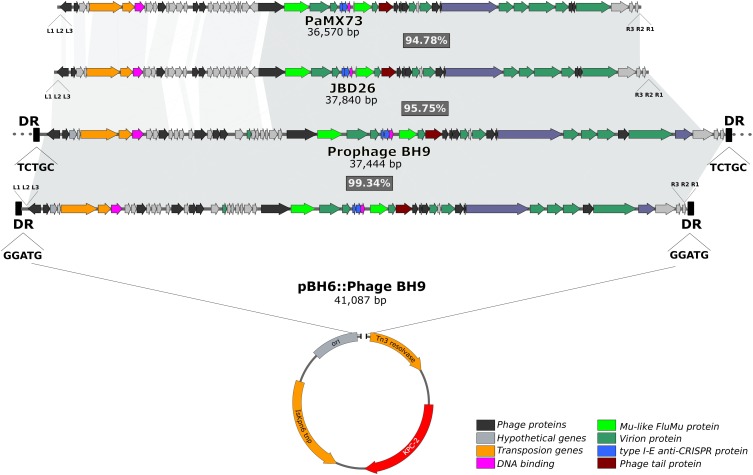
Comparisons of pBH6::Phage BH9 against its relative prophage inserted into *P. aeruginosa* BH9 main chromosome, and Mu phages PaMx73 and JBD26. The shared regions among the phages are highlighted in gray.

It has previously been reported that the *bla*_KPC-2_ gene in *P. aeruginosa* BH6, which was isolated from the same nosocomial environment, was harbored by a small plasmid pBH6 (3,652 kb) (Galetti et al., 2016). Indeed, comparative genomic analysis of the assembled *P. aeruginosa* BH9 genome revealed that the *bla*_KPC-2_ gene was included in an additional and larger contig containing identical sequence to the pBH6 plasmid, and also encoding several additional phage-related genes (hereafter called prophage BH9). Interestingly, dot plot analysis indicated that the beginning and end of this additional contig contains the same sequence (∼7.2 kb of overlap), strongly indicating for a circular structure. *In silico* analyses showed that prophage BH9 was a Mu-like phage, containing all well-known hallmarks of Mu-like phages (Cazares et al., 2014; Harshey, 2014), such as the transposition and structural genes, the triplet 5′-TGT at the phage ends, the transposase binding sites at the left (L1, L2, and L3) and right (R1, R2, and R3) ends, and 5-bp direct repeats (GGATG) flanking the insertion site at the pBH6 backbone. These findings strongly indicates that the prophage BH9 was inserted into the plasmid pBH6, creating the co-integrate pBH6::Phage BH9 phage-like plasmid (Figure 1). After circularizing and trimming procedures, the recovered phage-like plasmid structure exhibited 41,087 bp carrying the BH9 Mu-like prophage and the pBH6. Furthermore, it was also noted that the BH9 Mu-like prophage was also inserted on the *P. aeruginosa* BH9 chromosome, exhibiting a distinct 5-bp direct repeats (TCTGC), but not containing the pBH6 sequence and the *bla*_KPC-2_ gene (Figure 1). The presence of both, BH9 Mu-like prophages, strongly indicates for a previous event of mobilization of the BH9 Mu-like phage by its replicative transposition mechanism, directly molding the *P. aeruginosa* BH9 genome and impacting the *bla*_KPC-2_ mechanism of propagation by the small plasmid pBH6 (Galetti et al., 2016).

In addition, using the PHASTER web server, three other different prophage regions (32,3 kb; 54,6 kb, 49,8 kb) were found in the chromosome of the BH9 isolate; however, none of them were related to Mu-like phages nor carrying additional cargo components, like as non-phage genes related to bacterial adaptation or virulence.

Moreover, comparative analysis with similar Mu-like phages available on the public genome databases revealed that the prophage BH9 showed 86% of coverage and 96% of identity with phage JBD26 and 82% of coverage and 97% of identity with PaMx73, where the main differences were related to phage genes related to the regulation of transposition (Figure 1). The high level of identity shared between the phage head and tail genes of the prophage BH9, JBD26, and PaMx73 may support for similar virion structure. PaMx73 was isolated from an environmental water sample (Cazares et al., 2014), whereas JBD26 was isolated from the Human Microbiome Project. Moreover, in Mu-like phages such as JBD26 and PaMx73, three imperfect repeat sequences (22 bp conserved putative transposase binding sites, L1, L2, L3 and R1, R2, R3) are located close to both genome ends (Harshey, 2014; Figure 1). Phage BH9 presented the 3 imperfect repeats L1 (GTGACTGTTTTGACACATGCCA), L2 (GGGAGTTTGGAAAAAATCGA), and L3 (AGCGCGGAATGAAAGATATTGA), located at the left end, at positions 10, 93, and 124 downstream from the 5′-TGT triplet, respectively, and also the R1 (CGCGCCAAATTTCGCGCCGC), R2 (CGCAGCCGGAACGGCTAGGGCG), and R3 (CGATGGCGATATTGTCGTCCTG) repeats at the right end, at positions 4, 46, and 93 upstream from the 5′-TGT triplet, respectively (Figure 1). In addition, the *c* repressor protein (which represses the lytic cycle and allows stable integration of the phage on the bacterial genome) was identified in both prophages and pBH6::Phage BH9 phage-like plasmid. Collectively, these results led us to suppose that the prophage BH9 encode the necessary traits to transpose and propagate in favored conditions.

In the last few years, many studies have focused on the role of phages as potential candidates for the spread of antibiotic resistance genes, since they can become vehicles for horizontal gene transfer and recombination (Pallen and Wren, 2007; Colavecchio et al., 2017a). Phage particles carrying antimicrobial resistance genes such as *bla*_CTX-M_, *bla*_SHV_, *bla*_TEM_, and *qnr* have already been identified in wastewater treatment plants, hospital effluents, and human feces and sputum from cystic fibrosis patients (Muniesa et al., 2004; Rolain et al., 2011; Balcazar, 2014; Jaque et al., 2018); however, the phage family and complete sequence are not yet available. Conversely, phage-like plasmids carrying antibiotic resistance genes, such as *bla*_CTX-M-15_ isolated from *Escherichia coli* and *mcr-1* from *Klebsiella pneumoniae*, were recently described (Colavecchio et al., 2017b; Zhou et al., 2018), demonstrating the emergence of these elements as a new platform for lateral gene transfer and antibiotic resistance gene exchange.

In the genomic era, Mu-like prophages have been found in multiple, distantly related species, indicating that they are widespread mobile genetic elements that play an important role shaping their host genomes. The findings of the present study suggest that pBH6 has captured the Phage BH9, creating the co-integrate pBH6::Phage BH9 and this phage-plasmid complex may represent a novel case of a phage-like plasmid. This study also shed new insights into the impact of the Mu-like phages into the vehicles of the dissemination of antimicrobial resistance genes in the *Pseudomonas* genus.

## Accession Numbers

The sequence of *P. aeruginosa* BH9 and pBH6::Phage BH9 were deposited at DDBJ/ENA/GenBank under the accession numbers CP029713 and CP029714, respectively. Accession numbers used were YMC11/02/R656 NC_028657; JBD24 NC_020203; D3 NC_002484; F10_NC_007805; JBD26 JN811560, and PaMx73 JQ067085.

## Ethics Statement

The *Pseudomonas aeruginosa* strain used in this study was isolated from a nasal swab of an inpatient at University Hospital of Belo Horizonte in 2011. The bacterium was isolated and identified as part of hospital procedures and donated to *Laboratório Especial de Bacteriologia e Epidemiologia Molecular* (LEBEM).

## Author Contributions

RG conceptualized and designed, performed acquisition, analyzed and interpreted the data, drafted the manuscript, and performed critical revision. LA interpreted the data, drafted the manuscript and performed critical revision. AV analyzed and interpreted the data, drafted the manuscript and performed critical revision. AD drafted the manuscript and performed critical revision.

## Conflict of Interest Statement

The authors declare that the research was conducted in the absence of any commercial or financial relationships that could be construed as a potential conflict of interest.

## Footnotes

1 http://pubmlst.org/paeruginosa/

2 https://cge.cbs.dtu.dk/services/MLST/

3 https://cge.cbs.dtu.dk/services/

4 https://cge.cbs.dtu.dk/services/ResFinder/
